# Oral health-related quality of life and associated factors among a sample from East China with severe early childhood caries: a cross-sectional study

**DOI:** 10.1186/s12903-023-03560-4

**Published:** 2023-11-07

**Authors:** Lianyi Yang, Shimin Zhao, Yuanbing Zhu, Guangyun Lai, Jun Wang

**Affiliations:** Department of Pediatric Dentistry, Shanghai Ninth People’s Hospital, College of Stomatology, National Center for Stomatology, Shanghai Key Laboratory of Stomatology, Shanghai Jiao Tong University School of Medicine, Shanghai Jiao Tong University, National Clinical Research Center for Oral Diseases, No. 500 Quxi Road, Huangpu District, Shanghai, China

**Keywords:** Early childhood caries, Oral health, Quality of life, Family impact, Child impact, Associated factors

## Abstract

**Background:**

To investigate the oral health-related quality of life (OHRQoL) and associated factors among a sample from East China with severe early childhood caries (S-ECC).

**Methods:**

A total of 316 children with S-ECC and their parents were recruited to participate in a cross-sectional study. Children were examined for caries status using criteria proposed by World Health Organization (WHO). The accompanying parent was required to provide demographic information and complete two validated questionnaires in Chinese: the early childhood oral health impact scale (ECOHIS) and the 5-item oral health impact profile (OHIP).

**Results:**

The study had a 98.1% response rate. Finally, the data of 300 children and their parents were analyzed. Mothers cared for their children far more than fathers in the included family (78.7% mother, 21.3% father). The mean age of children was 4.1 ± 0.7 years, ranging from 3 to 5. The mean dmft score was 13.8 ± 3.8. Few (13.7%) children never had a toothache. ECOHIS scores ranged from 0 to 38, with a mean score of 16.2 ± 7.2. The mean OHIP score was 2.9 ± 2.7. The parental age, family income, residence, history of pain, the dmft scores and parents’ OHIP showed associations with ECOHIS scores or domain scores (*P* < 0.05). The multiple regression analysis showed that the history of pain, accompanying parents’ OHIP, and the dmft scores were mainly associated with ECOHIS and child impact (*P* < 0.05); parental age was associated with family impact (*P* = 0.024).

**Conclusions:**

The parent’s OHRQoL was associated with the children’s OHRQoL, indicating that policymakers and clinical practitioners should improve both children’s and their parents’ oral health. Furthermore, the caries severity and the history of dental pain impacted children’s OHRQoL.

## Introduction

Dental caries is one of the most common chronic diseases affecting children worldwide [1]. Caries in the primary dentition of young children can be classified as early childhood caries(ECC) or severe early childhood caries (S-ECC) [2]. According to the 4th Chinese National Oral Health Survey, the caries prevalence was 50.8%, 63.6% and 71.9%, and the mean decayed-missing-filled teeth (dmft) was 2.28, 3.40 and 4.24 for 3-, 4- and 5-year-old children, respectively [3]. Due to the increased prevalence from 66 to 71.9% of 5-year-old children in the past decade, ECC is an ongoing oral health issue among Chinese preschool children, bringing a heavy burden to society and an acute need for attention [3].

Both ECC and S-ECC result from the interaction of multiple factors, such as cariogenic microorganisms, exposure to fermentable carbohydrates through inappropriate feeding practices, and various social variables [4]. Beyond the associated pain and suffering, ECC, especially left untreated, can have long-term adverse health outcomes for children [2, 5]. It can affect overall physical growth and development due to poor nutrition and lead to poor academic performance or reduced learning ability because of lost school days. Moreover, ECC affects parents indirectly, leading to family stress, disrupted sleep, work loss and financial harm because of the time and money spent caring for their children. ECC, therefore, impacts children’s oral health-related quality of life (OHRQoL) [6]. A previous meta-analysis found that compared with children without caries, children with at least one decayed tooth had poorer OHRQoL, and preschoolers with a dmft ≥ 6 had even poorer OHRQoL, implying the association between caries and negative impact on children’s OHRQoL tends to increase when the disease severity worsens [7]. Moreover, a significant difference in OHRQoL was found between children with ECC and S-ECC [8]. Therefore, S-ECC is more likely to affect the life quality of children and their families theoretically.

The impact of ECC on OHRQoL is evaluated using standardized scales such as the Early Childhood Oral Health Impact Scale (ECOHIS). This scale was developed by Pahel et al. to assess the impact of oral and dental health problems on the life quality of children aged 6–14 [9]. Subsequently, it has been translated into various languages and applied to children younger than six years old worldwide [10–13]. A Chinese version of ECOHIS showed high validity and reliability when applied to children aged five or younger [14]. Until now, research conducted in mainland China mainly focused on the OHRQoL of preschoolers from a local region [15] and the change in OHRQoL of children with caries following dental treatment under general anesthesia [16, 17]. However, the OHRQoL of children with S-ECC, who carry the most considerable burden of dental caries and need understanding, have yet to be investigated. Besides, as parents play a pivotal role in children’s development and oral health care [18], whether there is a relation between children’s OHRQoL and parents’ OHRQoL has not been evaluated. Given the paucity in this field, this cross-sectional study aimed to evaluate the OHRQoL of Chinese preschool children with S-ECC and to identify the factors associated with the OHRQoL of these children. We hypothesized that parents’ OHRQoL was associated with the OHRQoL of their children with S-ECC.

## Materials and methods

### Study design and sample selection

This cross-sectional study was conducted between April and November 2021 in the Department of Pediatric Dentistry, Shanghai Ninth People’s Hospital, Shanghai Jiao Tong University School of Medicine. Before initiating the study, the sample size was determined using the Power Analysis & Sample Size (PASS) software 16.0 with a 95% confidence interval, a standard deviation of 7.9 (the standard deviation of ECOHIS score from a Hongkong sample with S-ECC [19]), a precision of 1, and a 20% non-response rate. The minimum sample needed was 300.

All the Children who attended our department and met the criteria were invited. The inclusion criteria were children aged 3 to 5, diagnosed with S-ECC, and classified as class I of the American Society of Anesthesiologists Physical Status (ASAP). A dentist explained the study information to the parents. If the parents were illiterate or unable to understand the survey, their children would be excluded.

The Ethics Committee of Shanghai Ninth People’s Hospital approved the survey protocol (No. SH9H-2020-T191-1). Participation was voluntary. Written informed consent was obtained from parents before the survey.

### Clinical examination

According to the American Academy of Pediatric Dentistry (AAPD), the presence of 1 or more decayed (non-cavitated or cavitated lesions), missing (due to caries), or filled tooth surfaces (dmfs) of any primary tooth in a child younger than six years old is classified as early childhood caries(ECC) [2]. Any sign of smooth-surface caries in children under three indicates severe early childhood caries (S-ECC). Moreover, from ages 3 through 5, the dmfs score of greater than or four (age three), greater than or equal to five (age four), or greater than or equal to six (age five) is also defined as S-ECC [2]. The presence of caries was assessed using the WHO 2013 criteria [20]. The same trained dentist examined all the children and recorded the pain history based on the parental complaint. Before the examination, parents were asked whether their children were experiencing any tooth-related pain. A positive or negative response would be recorded. The dentist examined children with a plane mouth mirror and a probe under artificial light. No radiographs were taken for this study. Since caries were most likely to be observed clinically in the posterior and maxillary anterior primary teeth among the included children, we classified the dmft scores into two subgroups of ≤ 14 and > 14 in this study.

### Questionnaire survey

After the dental examination for a child, the child’s parent was asked to complete a Chinese questionnaire comprising the child’s characteristics and socioeconomic background, the parental and child’s OHRQoL.

Children’s OHRQoL was assessed using ECOHIS in Chinese [14], which consists of 13 questions and is divided into the child impact section(CIS) and family impact section(FIS). Questions 1–9 form CIS comprising four domains: child symptom, child function, child psychology, and child self-image and social interaction. The four left questions constitute FIS covering two domains: parent distress and family function. Each question has five optional coded responses: 0 = never, 1 = hardly ever, 2 = occasionally, 3 = often, 4 = very often and 5 = don’t know. A total score is the sum of the graded questions. Questionnaires with ‘don’t know’ responses were excluded from the study.

Parents’ OHRQoL was evaluated using a 5-item Oral Health Impact Profile(OHIP), translated and validated in Chinese in 2020 [21]. The five items are the following questions:

1. Have you had difficulty chewing any foods because of problems with your teeth, mouth, dentures or jaw?
2. Have you had painful aching in your mouth?
3. Have you felt uncomfortable about the appearance of your teeth, mouth dentures or jaws?
4. Have you felt that there has been less flavor in your food because of problems with your teeth, mouth, dentures or jaws?
5. Have you had difficulty doing your usual jobs because of problems with your teeth, mouth, dentures or jaws?

Each item has five possible options and was coded: 0 = never, 1 = hardly ever, 2 = occasionally, 3 = fairly often, and 4 = very often. A total score ranges from 0 to 25.

### Data analysis

Categorical variables were expressed as frequencies and percentages (%). Continuous variables were exhibited with the mean and standard deviation (SD). If the distribution of continuous variables was normal, the differences between groups were evaluated using independent samples t-tests or one-way analysis of variance (ANOVA). Independent samples t-tests were used to compare mean scores between two groups with Leven’s tests calculating equality of variances. Welch t-tests were applied when unequal variances appeared. ANOVA was used to compare scores among three groups with Leven’s tests calculating equality of variances. Welch’s ANOVA were applied when unequal variances appeared. Otherwise, non-parametric tests were applied. A multiple linear regression model was used to explore the critical factors associated with ECOHIS. The model was adjusted for parent gender, parent age, mother’s education level, father’s education level, family income per month, residence, number of children, OHIP, child’s gender, child’s age, dmft score and history of pain. All the data were analyzed using SPSS Statistics 25.0 (IBM, Chicago, IL, USA). A p-value less than 0.05 was statistically significant.

## Results

A total of 316 children diagnosed with S-ECC and their parents participated in the study. Among the 316 recruited, 310 responses were complete (98.1% response rate). Ten child-parent dyads were excluded due to the ‘don’t know’ responses in the ECOHIS questionnaire. Finally, the data of the remaining 300 children (94.9%) were analyzed. The Cronbach’s alpha coefficients were 0.85 and 0.835, indicating the high reliability of the ECOHIS and OHIP scales.

Table 1 presents the characteristics of children and parents. Mothers cared for children far more than fathers in the recruited families (78.7% mother, 21.3% father). The mean age of accompanying parents was 34.8 ± 5.0 years(52.3% <35, 47.7% ≥35). Among all the parents, more than 60% of the accompanying parents’ education level was university or above. Most families (83.4%) had a monthly income above CNY 10,000. The residence was unequally distributed (78.3% Shanghai, 21.7% non-Shanghai). Almost half of all families (44.7%) tended to raise one child rather than more. The mean OHIP score was 2.9 ± 2.7. The gender of children was approximately equally distributed (54.3% male, 45.7% female). The mean age was 4.1 ± 0.7 years, ranging from 3 to 5. Each child had a dmft score of at least 6, with a mean score of 13.8 ± 3.8.

**Table 1:** Parent and child’s characteristics in the study (n = 300)

Parent and child’s characteristics	Frequency	Percentage(%)
*Parent’s demographics*		
Relationship to the child		
Mother	236	78.7
Father	64	21.3
Age (years)		
< 35	157	52.3
≥ 35	143	47.7
Mother’s education level		
College or below	116	38.7
University or above	184	61.3
Father’s education level		
College or below	115	38.3
University or above	185	61.7
Family income per month		
<CNY 9999	50	16.7
CNY 10000-19999	86	28.7
≥CNY 20,000	164	54.7
Residence		
Shanghai	235	78.3
Non-Shanghai	65	21.7
Number of children		
1	134	44.7
2	89	29.7
3	77	25.7
OHIP		
< 2	111	37.0
2–4	83	27.7
≥ 4	106	35.3
***Child’s demographics and caries status***		
Gender		
Male	163	54.3
Female	137	45.7
Age		
3	66	22.0
4	142	47.3
5	92	30.7
dmft		
≤ 14	183	61.0
>14	117	39.0
History of pain		
No pain	41	13.7
With pain	259	86.3

OHIP: oral health impact profile; CNY: Chinese Yuan

Table 2 describes the frequencies of 300 parents’ ECOHIS responses. ECOHIS scores ranged from 0 to 38, with a mean score of 16.2 ± 7.2. Only a few parents (13.7%) reported that their children never had dental-related pain. In the child function domain, 72.3% of parents reported the influence of S-ECC on children’s preschool, daycare or school work. For the child psychology domain, sleeplessness (62.3%) and irritation or frustration (65.0%) existed similarly. Children’s dental troubles brought more impact on the family than the child, especially parental distress. Around 90% of the parents felt upset (89.7%) and guilty (91.3%) because of their children’s dental problems or dental treatments. Regarding the family function, parents’ responses reflected more impact of taking time off work (85.7%) than money spent (78.3%).

**Table 2:** Responses to the Chinese Early Childhood Oral Health Impact Scale (ECOHIS) items (n = 300)

Impact	ECOHIS response, n (%)				
	Never	Hardly ever	Occasionally	Often	Very often
**Child impact section**					
***Child symptom***					
1.How often has your child had pain in the teeth, mouth or jaws?	41(13.7)	74(24.7)	150(50.0)	28(9.3)	7(2.3)
***Child function***					
2. How often has your child had difficulty drinking hot or cold beverages because of dental problems or dental treatments?	107(35.7)	94(31.3)	78(26.0)	19(6.3)	2(0.7)
3. How often has your child had difficulty eating some foods because of dental problems or dental treatments?	96(32.0)	90(30.0)	85(28.3)	23(7.7)	6(2.0)
4. How often has your child had difficulty pronouncing any words because of dental problems or dental treatments?	150(50.0)	105(35.0)	30(10.0)	10(3.3)	5(1.7)
5. How often has your child missed preschool, daycare or school because of dental problems or dental treatments?	83(27.7)	94(31.3)	106(35.3)	14(4.7)	3(1.0)
***Child psychology***					
6. How often has your child had trouble sleeping because of dental problems or dental treatments?	113(37.7)	106(35.3)	74(24.7)	6(2.0)	1(0.3)
7. How often has your child been irritable or frustrated because of dental problems or dental treatments?	105(35.0)	99(33.0)	86(28.7)	9(3.0)	1(0.3)
Self-image and social interaction					
8. How often has your child avoided smiling or laughing when around other children because of dental problems or dental treatments?	174(58)	88(29.3)	29(9.7)	7(2.3)	2(0.7)
9. How often has your child avoided talking with other children because of dental problems or dental treatments?	159(53.0)	94(31.3)	43(14.3)	4(1.3)	0(0.0)
**Family impact section**					
***Parental distress***					
10. How often have you or another family member been upset because of your child’s dental problems or dental treatments?	31(10.3)	39(13.0)	134(44.7)	75(25.0)	21(7.0)
11. How often have you or another family member felt guilty because of your child’s dental problems or dental treatments?	26(8.7)	23(7.7)	108(36.0)	110(36.7)	33(11.0)
***Family function***					
12. How often have you or another family member taken time off from work because of your child’s dental problems or dental treatments?	43(14.3)	73(24.3)	158(52.7)	23(7.7)	3(1.0)
13. How often has your child had dental problems or dental treatments that had a financial impact on your family?	65(21.7)	106(35.3)	108(36.0)	17(5.7)	4(1.3)

Table 3 shows the association between all the variables and ECOHIS scores. According to the statistical results, parent gender had no association with the total score or each domain, while parent age was associated with the ECOHIS score, family impact and parental distress domain. Younger parents reported higher scores for both family impact (*P* = 0.003) and parental distress (*P* = 0.001). Compared with mothers with an education level of college or below, those with higher education level reported significantly lower scores in the family impact section (*P* = 0.029) and family function domain (*P* = 0.033). On the other side, fathers’ education level showed no relation with ECOHIS scores. Parents from families with a monthly income of less than 10,000 reported the highest ECOHIS scores (*P* = 0.02), child symptoms scores (*P* = 0.006), family impact scores (*P* = 0.008) and family function scores (*P* < 0.0001). The residence was associated with ECOHIS (*P* = 0.024), child impact domain (*P* = 0.038) and child symptoms domain (*P* = 0.002). Girls scored significantly higher than boys in the child self-image domain (*P* = 0.014). Child age had no association with ECOHIS and each domain. Children with higher dmft scores presented higher scores in all the domains except the child symptoms and psychological domain. The history of pain was significantly associated with ECOHIS and the child impact section (*P* < 0.05). Parents’ OHIP scores had an association (*P* < 0.05) with every domain except the parent distress domain.

**Table 3:** Association between various parent and child characteristics and different domains of ECOHIS scores (mean, SD, n = 300)

Characteristics	ECOHIS	Child impact	Child symptoms	Child function	Child psychological	Child self-image	Family impact	Parental distress	Family function
Parent gender									
Mother	16.0(7.5)	8.7(5.3)	1.6(0.9)	4.1(2.7)	1.9(1.6)	1.1(1.4)	7.3(3.1)	4.4(2.0)	2.8(1.6)
Father	17.0(5.8)	9.7(4.7)	1.6(0.8)	4.5(2.4)	2.1(1.4)	1.5(1.5)	7.2(2.3)	4.3(1.7)	3.0(1.1)
Parent age	0.013*						0.003**	0.001**	
< 35	17.2(7.3)	9.4(5.1)	1.7(0.9)	4.4(2.6)	2.0(1.6)	1.3(1.6)	7.7(3.2)	4.7(2.0)	3.0(1.6)
≥ 35	15.1(6.9)	8.4(5.2)	1.5(0.9)	3.9(2.6)	1.9(1.6)	1.1(1.4)	6.7(2.5)	4.0(1.8)	2.7(1.3)
Mother’s education level							0.029*		0.033*
College or below	17.2(7.4)	9.5(5.3)	1.7(0.9)	4.5(2.7)	2.1(1.7)	1.3(1.6)	7.7(3.0)	4.6(1.9)	3.1(1.6)
University or above	15.5(7.0)	8.5(5.1)	1.6(0.9)	3.9(2.6)	1.8(1.5)	1.2(1.4)	7.0(2.9)	4.3(1.9)	2.7(1.4)
Father’s education level									
College or below	16.8(7.2)	9.3(5.3)	1.8(0.9)	4.5(2.7)	1.9(1.6)	1.2(1.5)	7.5(2.9)	4.5(1.9)	3.0(1.5)
University or above	15.8(7.1)	8.6(5.1)	1.5(0.9)	3.9(2.6)	1.9(1.6)	1.2(1.4)	7.1(2.9)	4.3(2.0)	2.8(1.4)
Family income per month	0.02*		0.006**				0.008*		< 0.001***
≤CNY 9999	18.4(6.8)	10.2(5.1)	2.0(1.9)	4,6(2.7)	2.3(1.5)	1.3(1.5)	8.2(2.7)	4.8(1.8)	3.4(1.5)
CNY 10000-19999	16.6(7.7)	9.2(5.3)	1.6(0.9)	4.2(2.7)	2.0(1.6)	1.4(1.5)	7.4(3.2)	4.3(2.0)	3.1(1.6)
≥CNY 20,000	15.3(6.9)	8.4(5.1)	1.5(0.9)	4.0(2.6)	1.8(1.6)	1.1(1.4)	6.9(2.8)	4.3(1.9)	2.6(1.4)
Residence	0.024*	0.038*	0.002**						
Shanghai	15.7(7.2)	8.6(5.1)	1.5(0.9)	4.0(2.6)	1.9(1.6)	1.2(1.5)	7.1(2.9)	4.3(2.0)	2.8(1.5)
Non-Shanghai	18.0(7.0)	10.1(5.2)	2.0(1.0)	4.6(2.8)	2.2(1.5)	1.3(1.5)	7.8(2.9)	4.7(1.9)	3.1(1.5)
Number of children									
1	16.5(7.1)	8.9(5.4)	1.6(0.9)	4.1(2.7)	1.9(1.7)	1.3(1.5)	7.6(2.7)	4.7(1.7)	2.9(1.4)
2	16.2(7.6)	9.2(5.2)	1.7(1.0)	4.2(2.7)	2.1(1.6)	1.3(1.6)	6.9(3.2)	4.2(2.1)	2.8(1.6)
3	15.5(6.9)	8.5(4.9)	1.7(0.8)	4.1(2.5)	1.7(1.4)	1.1(1.3)	7.0(3.0)	4.1(2.0)	2.8(1.4)
Child gender						0.014*			
Male	15.9(7.4)	8.7(5.3)	1.6(1.0)	4.2(2.7)	1.8(1.5)	1.0(1.4)	7.3(3.1)	4.4(2.0)	2.8(1.5)
Female	16.4(6.9)	9.2(5.1)	1.6(0.8)	4.1(2.5)	2.0(1.7)	1.4(1.5)	7.3(2.8)	4.4(1.9)	2.9(1.4)
Child age									
3	16.1(8.4)	8.7(5.9)	1.6(1.0)	3.9(3.0)	1.9(1.8)	1.3(1.5)	7.4(3.6)	4.6(2.3)	2.9(1.8)
4	16.3(6.8)	9.1(4.9)	1.7(1.0)	4.3(2.6)	1.9(1.5)	1.2(1.4)	7.2(2.7)	4.4(1.8)	2.8(1.4)
5	16.0(6.8)	8.8(5.2)	1.6(0.8)	4.0(2.5)	2.0(1.5)	1.2(1.5)	7.2(2.7)	4.3(1.9)	2.9(1.4)
dmft	0.002**	0.005**		0.003**		0.049*	0.013*	0.046*	0.048*
≤ 14	15.2(7.4)	8.2(5.2)	1.6(1.0)	3.8(2.6)	1.9(1.6)	1.1(1.3)	6.9(3.0)	4.2(2.0)	2.7(1.5)
>14	17.7(6.6)	10.0(5.1)	1.7(0.8)	4.7(2.6)	2.0(1.6)	1.5(1.6)	7.8(2.7)	4.7(1.8)	3.1(1.4)
History of pain	< 0.001***	< 0.001***	< 0.001***	< 0.001***	< 0.001***	0.003**			
No pain	10.5(6.1)	4.0(4.1)	0.0(0.0)	2.5(2.8)	0.8(1.2)	0.7(1.3)	6.6(3.3)	4.0(2.2)	2.6(1.6)
With pain	17.1(6.9)	9.7(4.9)	1.9(0.7)	4.4(2.5)	2.1(1.6)	1.3(1.5)	7.4(2.9)	4.5(1.9)	2.9(1.4)
OHIP	< 0.001***	< 0.001***	0.041*	< 0.001***	< 0.001***	0.006**	0.021*		0.037*
< 2	14.1(8.0)	7.5(5.6)	1.5(1.0)	3.4(2.7)	1.6(1.7)	1.0(1.5)	6.6(3.2)	4.0(2.1)	2.6(1.6)
2–4	16.2(6.3)	8.6(4.7)	1.7(0.9)	4.1(2.4)	1.8(1.5)	1.1(1.4)	7.6(2.9)	4.5(1.8)	3.0(1.4)
≥ 4	18.3(6.3)	10.6(4.7)	1.8(0.8)	4.9(2.5)	2.4(1.4)	1.5(1.5)	7.7(2.5)	4.7(1.8)	3.0(1.3)

ECOHIS:early childhood oral health impact scale; OHIP: oral health impact profile;CNY: Chinese Yuan

*P < 0.05; **P < 0.01; ***P < 0.001

As shown in Table 4, the multiple regression analysis exhibited that both the dmft scores and parents’ OHIP scores were significantly associated with ECOHIS scores, child impact and family impact (*P* < 0.05). Moreover, the history of pain showed an association with both ECOHIS scores and child impact (*P* < 0.001). Parent age was significantly associated with family impact (*P* = 0.024).

**Table 4:** Multiple regression analysis: ECOHIS scores for the parent and child characteristics in the study (n = 300)

Variable	Unstandardized coefficient	Standard error	*P*-Value
ECOHIS (adjusted R^2^ = 0.169)			
History of pain	5.017	1.150	< 0.001***
dmft	2.209	0.792	0.006**
OHIP	1.846	0.465	< 0.001***
Child impact (adjusted R^2^ = 0.191)			
History of pain	4.72	0.821	< 0.001***
dmft	1.457	0.565	0.01*
OHIP	1.269	0.332	< 0.001***
Family impact (adjusted R^2^ = 0.070)			
Parent age	-0.774	0.341	0.024*
dmft	0.753	0.342	0.029*
OHIP	0.577	0.201	0.004**

ECOHIS: early childhood oral health impact scale; OHIP: oral health impact profile

*P < 0.05; **P < 0.01; ***P < 0.001

## Discussion

The present study assessed the oral health-related quality of life among a sample of preschoolers with S-ECC and associated factors. The results showed that the history of pain, and the dmft scores were mainly associated with children’s ECOHIS and child impact, and parental age was associated with family impact. Moreover, the association between the parent’s OHRQoL and children’s OHRQoL found in this study supported our hypothesis.

Previous studies have found a significant difference in OHRQoL between children with and without caries and a definite negative impact of ECC on the OHRQoL of children [6, 7, 19, 22, 23]. However, this study mainly focused on the OHRQoL of children with S-ECC due to the paucity of related research in mainland China. Regarding the sample, all the included children had a severe caries status. The mean dmft score of 13.8 is higher than the mean score of 10.2 among children with S-ECC in a similar study from Hong Kong, China [19]. This may be because Shanghai Ninth People’s Hospital is famous for Dentistry in East China; many patients from the Department of Pediatric Dentistry were referred by dental practitioners from other hospitals or clinics for more specialized dental care.

In order to explore factors associated with the OHRQoL of children with S-ECC, this study drew on previous studies on the OHRQoL of children with ECC and included social-economical parameters, parent gender and age, dmft score and history of pain as analysis variables [7, 8, 15]. Moreover, the association between parents’ and children’s OHRQoL was investigated. Evidence from the literature has shown that the ECC severity was related to children’s OHRQoL, including impacts on the child and family [7]. The present study also demonstrated that the increased S-ECC severity led to higher scores of total ECOHIS and child and family impact sections.

In 2017, Patrick Hescot, president of the FDI World Dental Federation, addressed that oral health, as a fundamental component of health and physical and mental well-being, is a multifaceted entity. It includes the ability to confidently do a series of skills (speak, smile, smell, taste, touch, chew, swallow, and convey emotions through facial expressions) without pain, discomfort, or disease of the craniofacial complex [24]. It reflects the physiological function, status, and psychosocial function essential to the quality of life. OHRQoL assesses the impact of oral health problems on an individual’s behaviour and social functioning and complements the conventional clinical assessment of oral health [24]. Several OHRQoL instruments have been recently developed for children [25]. ECOHIS, applied in this study, is the most commonly used scale for children under six.

As oral health is influenced by the person’s changing experiences, perceptions, expectations, and ability to adapt to changing circumstances [26], OHRQoL evaluation is subjective to a certain extent. Due to their young age, children may be unable to interpret questions and a long-term view of events precisely and patiently. Therefore, while not ideal, a proxy by the parent may be an acceptable and useful alternative [27]. In order to ensure the accuracy of information provided by parents and to avoid the “don’t know” responses to the ECOHIS questionnaire as much as possible, the parents in this study were the primary caregiver for the children. The parent gender distribution in the study implied that far more mothers cared for children than fathers in mainland China.

The results showed a significant association between the parent age and the ECOHIS scores and family impact, which is consistent with the finding that the greater age of the mother had a positive impact on the child’s OHRQoL reported by Martins-Júnior et al. [6]. It may be because younger parents feel less secure or have less experience in caring for their children and are easier to feel guilty because of their children’s dental problems [6], leading to an increase in ECOHIS and family impact scores. However, other studies did not demonstrate the association between parental age and ECOHIS scores [22, 28]. This controversy indicates that the influence of parent age on ECOHIS and family impact needs further study.

Family socioeconomic status, usually evaluated by family income and education level, affecting parental perceptions regarding their children’s oral health, were significant predictors of children’s OHRQoL [29]. Children from high-income families were more likely to have better OHRQoL, which was also verified in this study. It is assumed that children from high-income families have more access to health care and prevention, which might lead to a better quality of life. However, regarding the relation of parental education level to children’s OHRQoL, our study found that the education level of neither the mother nor the father was associated with the total ECOHIS scores. The statistical analysis only showed the impact of the mother’s education level on the FIS. It may be because the caries status of this sample was so severe that the ECOHIS score was relatively high; the influence of the mother’s education might be diminished. On the other hand, because the primary caregivers of most children were mothers, the influence of their education level is embodied in the family impact section. Furthermore, residence showed an association with children’s OHRQoL. Shanghai, one of the most developed cities in China, has better medical conditions and services for local people [30]. Besides, a national investigation in 2018 showed that the ratio of stomatologists/dentists to the population in Shanghai was 1:5,409, which was higher than the number in the whole country, which was 1:7,768 [31]. It is reasonable that children living in Shanghai have relatively better OHRQoL than those from other places, as shown in this study.

ECC has many risk factors. Parental knowledge and behaviors are the most concerned factors due to the potential to be altered, thus improving children’s oral health [32]. Although it has been neglected previously, it is noteworthy that both bivariate and multivariate analysis showed a significant association of parents’ OHRQoL with both children’s OHRQoL, child impact and family impact section among this sample. It intensely implies that the oral health education and prevention strategies regarding ECC by either clinical practitioners or governmental programs in China should focus on not only the children’s oral hygiene and health, but also the parental oral health, or even the families’ oral health status.

Research suggests that children as young as three years old can have body image issues [33]. ECC, if untreated, with defective brown or dark teeth can impact the children’s psychological health with the shyness of opening their mouth when speaking or smiling. Although neither child age nor gender was related to the total ECOHIS scores, child gender was associated with the child self-image domain. Girls had a remarkably higher score for the child self-image than boys in this study, indicating that girls may suffer more from the impact of caries on the self-appearance than boys.

Dental pain, one of the most common symptoms because of untreated decayed teeth, such as pulp involvement and abscess, is reported to be a primary reason for seeking dental treatment in early childhood [34]. Nearly 90% of the included children had a pain history. Consistent with other studies finding that a history of dental pain indicates poor OHRQoL in the child [19, 35], the history of pain was significantly associated with the higher scores of ECOHIS and child impact section in this study. According to the Chinese National Epidemiological Survey in 2017, 97% of children aged 5 with decayed primary teeth received no treatment [3]. Therefore, in addition to caries prevention, oral health education and programs should encourage parents to bring their children to see dentists from a young age. Moreover, the government may have to make strategies to provide more medical services for treating children’s decayed primary teeth, avoiding the negative impact on their quality of life due to dental pain and other complications.

The present study suffered from limitations. First, due to the cross-sectional study design, casual relationships cannot be proved in this study. Second, as we recruited children and parents from a single hospital, it is impossible to reflect a wide national range of children. Additionally, since all the included children attended to seek dental treatment, we assumed that these children had a worse quality of life than those who had not presented, leading to some bias in our results. Large-scale of studies or longitudinal studies are needed to verify the findings and clarify the controversies in this study.

## Conclusions

This study showed an association between the parent’s OHRQoL and children’s OHRQoL, indicating that policymakers and clinical practitioners should improve both children’s and their parents’ oral health. Furthermore, children’s OHRQoL worsened with the presence of pain and higher dmft cores. More effort is needed to encourage parents to pay more attention to children’s oral health.

## Data Availability

All data collected and analyzed in this research are demonstrated in the tables or available from the corresponding author on reasonable request.

## Abbreviations

S-ECC: Severe early childhood caries
OHRQoL: Oral health-related quality of life
WHO: World Health Organization
ECOHIS: Early childhood oral health impact scale
OHIP: Oral health impact profile
AAPD: American Academy of Pediatric Dentistry
ECC: Early childhood caries
PASS: Power Analysis & Sample Size
ASAP: American Society of Anesthesiologists Physical Status
CIS: Child impact section
FIS: Family impact section
SD: Standard deviation
ANOVA: One-way analysis of variance
